# Comprehension by Caregivers and Adolescents of Clinical Trial Information Delivered via Multimedia Video Versus Conventional Practice: Nonrandomized Controlled Trial

**DOI:** 10.2196/44252

**Published:** 2023-06-22

**Authors:** Kathryn V Blake, Holly Antal, H Timothy Bunnell, Jiaxian He, Robert Henderson, Janet T Holbrook, Suzanne M McCahan, Chris Pennington, Linda Rogers, David Shade, Elizabeth A Sugar, Alexandra Taylor, Robert A Wise, Tim Wysocki

**Affiliations:** 1 Center for Pharmacogenomics and Translational Research Nemours Children's Health Jacksonville, FL United States; 2 Division of Psychology and Neuropsychology Nemours Children's Health Jacksonville, FL United States; 3 Bioinformatics Research Informatics Center Nemours Children's Health Wilmington, DE United States; 4 Center for Clinical Trials and Evidence Synthesis Johns Hopkins Bloomberg School of Public Health Baltimore, MD United States; 5 Mount Sinai-National Jewish Health Respiratory Institute Icahn School of Medicine at Mount Sinai New York, NY United States; 6 Center for Healthcare Delivery Science Nemours Children's Health Jacksonville, FL United States; 7 Johns Hopkins Pulmonary and Critical Care Medicine Johns Hopkins University School of Medicine Baltimore, MD United States

**Keywords:** adolescent, clinical trial, comprehension, informed consent, internet, multimedia

## Abstract

**Background:**

Research participants often misunderstand the required elements of informed consent information, whether provided in written or oral format. Informed consent instruments with embedded evidence-based learning theory principles administered in multimedia electronic formats may improve comprehension and retention.

**Objective:**

This study aims to determine whether study information comprehension and retention using an interactive multimedia video consent process was noninferior to comprehension and retention after an in-person face-to-face interaction with a conventional written consent document for caregivers and adolescents enrolled in a clinical trial.

**Methods:**

Participants were caregivers and children aged 12 to 17 years who were enrolled in a clinical trial of asthma treatment. Consent information was presented as a multimedia web-based video consent interaction or as a conventional written consent document with in-person interaction between the prospective participants and the study staff. The trial used a parallel nonrandomized noninferiority design that compared the 2 consent methods. Caregivers and adolescents completed a 17-item open-ended comprehension questionnaire (score range 17-51) at enrollment and at the end of the study 20 weeks later. Comprehension and retention were compared between the consent formats. Noninferiority was established if the 95% CI upper bound of the difference in scores (conventional format minus web-based) was less than the noninferiority margin of 2.4; superiority was established if the upper bound of the CI was <0.

**Results:**

In total, 54 caregiver and adolescent dyads completed the interactive multimedia web-based video consent, and 25 dyads completed the conventional consent. Overall, 33% (26/79) of all adolescents were Black, 57% (45/79) were male, and 61% (48/79) had a household income of <US $60,000 per year. For caregivers, the interactive multimedia web-based format was noninferior to the conventional format at enrollment (difference between the conventional and web-based formats: mean −0.30, 95% CI −2.52 to 1.92) and was superior at the end of the study 20 weeks later (mean −2.20, 95% CI −3.9 to −0.5). There was a loss of comprehension over 20 weeks (mean −1.65, 95% CI −3.1 to −0.19) with the conventional format but not with the multimedia web-based format (mean 0.14, 95% CI −0.84 to 1.12). For adolescents, the noninferiority of the multimedia web-based format was not established.

**Conclusions:**

Caregivers who are considering enrolling their adolescent in an asthma clinical trial have similar comprehension of study information when delivered through an interactive multimedia web-based platform, which incorporates evidence-based learning theory principles, compared with having a conventional in-person, face-to-face discussion. The retention of study information over time was better with the multimedia format for caregivers.

**Trial Registration:**

ClinicalTrials.gov NCT02061280; https://clinicaltrials.gov/ct2/show/NCT02061280 and NCT01437995; https://clinicaltrials.gov/ct2/show/NCT01437995

## Introduction

### Background

Obtaining informed consent is one of the main protections of human subject participation in research and must be obtained before any research procedure, unless a waiver is approved by an institutional review board (IRB) [1]. The consent process is characterized by three features: (1) disclosing sufficient information for the participant to make an informed decision, (2) facilitating the understanding of that information, and (3) underscoring the voluntariness of participation in research [1]. Enrolling dependent youth or child participants in research studies adds complexity because of the need to obtain permission from the parent or legal guardian as well as assent from the child (as young as 7 years) who is capable based on age, maturity, and psychological state [2-4]. Federal regulations and guidance documents are silent on the information to be provided during the assent process; however, the assent would be expected to increase in complexity as a child ages. The provision of assent by a child expresses their willingness to participate, although there is no regulatory requirement to ensure that the required elements of consent have been effectively communicated [1,4]. Federal regulations allow informed consent information to be presented in a written or verbal format.

There is ample evidence that research participants have a poor understanding of the federally required elements in informed consent documents, regardless of format [5-15]. The problem is exacerbated by the complexity and length of typical written consent documents that now, because of intensified institutional oversight, have more regulatory requirements that require the inclusion of difficult-to-understand legal language [13,16]. Strategies to promote the understanding of research study information such as modified layouts, lower reading levels, and multimedia presentations have shown mixed results [16]. However, many of these strategies did not incorporate evidence-based learning principles [13,17,18]. Furthermore, studies that measured understanding often used closed-ended questions that primarily test recall versus open-ended questions, which can assess the comprehension of learned information. Few studies have assessed the comprehension of study information in children or adolescents who were actually enrolled in a clinical trial [12-14,19].

### Objectives

To address these issues, we designed an interactive multimedia video and associated website for caregivers and adolescents considering participation in an asthma clinical trial to compare study comprehension with the conventional in-person face-to-face consent process. The interactive multimedia video and website applied evidence-based learning theory principles designed to enhance learning while reducing cognitive load in participants with varying levels of health literacy [18,20-22]. We hypothesized that study comprehension following multimedia video consent and website interaction would be noninferior to the conventional consent process.

## Methods

### Overall Trial Design

**Figure 1:**
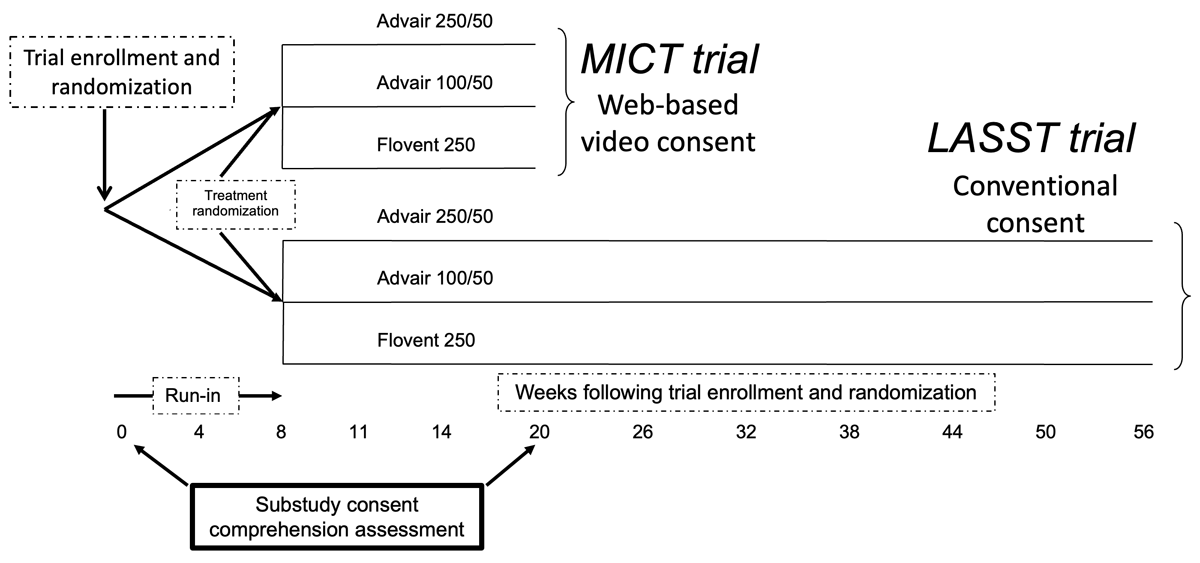
Study schema for conventional (Long-Acting Beta-Agonist Step-Down Study [LASST]) and web-based (Use of Mobile Devices and the Internet to Streamline an Asthma Clinical Trial [MICT]) trials. Consent comprehension was conducted at enrollment and 20 weeks later.

The study comprehension evaluation by consent format was designed as a substudy nested within 2 asthma clinical trials: one trial using conventional procedures for consent, enrollment, and follow-up (Long-Acting Beta-Agonist Step-Down Study [LASST]) and the other trial using web-based and video procedures for study conduct (Use of Mobile Devices and the Internet to Streamline an Asthma Clinical Trial [MICT]; Figure 1). The LASST clinical trial was a multicenter, double blinded to treatment (Advair 250/50 [GlaxoSmithKline], Advair 100/50 [GlaxoSmithKline], and Flovent 250 [GlaxoSmithKline]) study designed to evaluate de-escalation strategies in participants aged ≥12 years with moderate persistent asthma that was well controlled with a fixed-dose combination of inhaled corticosteroid plus a long-acting β_2_-agonist (LASST; NCT01437995) [23]. LASST included an 8-week run-in period before treatment randomization and involved 12 visits over 56 weeks. LASST was conducted from February 2012 to July 2015 at 18 network sites of the American Lung Association Airways Clinical Research Centers (ACRC) network [23]. The ACRC study sites are pulmonology and allergy subspecialty clinics within academic medical centers that have large racially and socioeconomically diverse patient populations.

The web-based trial with video procedures was designed to evaluate methods to reduce the burden of research participation (MICT; NCT02061280), was modeled after LASST with identical treatment groups but with only 2 on-site study visits (to obtain and return study equipment) and 4 web-based visits (using an iPad [Apple Inc] with FaceTime [Apple Inc]) over 12 weeks, and was conducted in adolescents aged 12 to 17 years [20,21]. The MICT trial was conducted in adolescents because the lead site was a pediatric institution. MICT was conducted at 6 of the 18 ACRC network sites that had study coordinators experienced in pediatric clinical trial research from November 2013 to February 2017.

A substudy to evaluate the comprehension of the conventional in-person, face-to-face consent format (in LASST participants) versus the multimedia web-based delivery process (in MICT participants) was conducted among adolescents and their caregivers at the 6 ACRC network pediatric sites by coordinators enrolling participants for both the LASST and MICT trials. Coordinators were trained and knowledgeable in the trial procedures, including obtaining informed consent from parents or guardians (hereafter referred to as caregivers) and adolescents. At the start of enrollment for the substudy, participants were randomized by trial type (LASST or MICT), and caregivers and adolescents were unaware of both trials when randomization was performed. After enrollment was completed in the LASST trial, all eligible participants were then enrolled in the MICT trial.

The details of the LASST and MICT trials and a description of the learning principles used in the development of the interactive multimedia video consent and website for the MICT trial have been previously described [20,21,23]. This paper presents a comparison of the consent procedures with respect to study *comprehension* between the LASST and MICT trials.

### Ethics Approval

The LASST and MICT trials were approved by the IRB at Nemours Children’s Health as research involving greater than minimal risk but presenting the prospect of direct benefit to the individuals per Code of Federal Regulations 45CFR46.405 and 21CFR50.52. Parental permission and assent were obtained from the legal caregivers and adolescents aged 12 to 17 years, respectively, and informed consent was obtained from adults aged ≥18 years. IRB approval numbers were #288148 (LASST) and #332965 (MICT).

### Enrollment and Payment for Participation

Participants enrolled in the LASST and MICT trials had well-controlled asthma (Asthma Control Test score of ≥20) [24], were on a medium dose of fluticasone-salmeterol combination inhaler (Advair, GlaxoSmithKline) for at least 4 weeks, and had less than a 10 pack-year smoking history with no smoking in the previous year [20,23].

On enrollment in the LASST or MICT trial, the participant was assigned a unique alphanumeric code and a name code that used the first and middle initials and the first 3 letters of the last name. The links to the codes were stored in password-protected computer files at the study site institution, with access limited to the research study team at the study sites. Data without personal identifiers were analyzed by the Center for Clinical Trials and Evidence Synthesis at Johns Hopkins Bloomberg School of Public Health.

Participants in the LASST trial were paid up to US $825 prorated over the course of 56 weeks for completing 11 study site visits. Participants in the MICT trial were paid up to US $430 prorated over the course of 20 weeks for completing 6 study visits (remote and on site) and each morning (US $1) and evening (US $1) diary card. Participants in the LASST trial were paid US $50 for the enrollment visit at which informed consent was obtained and study procedures were performed; participants in the MICT trial were paid US $25 for completing the multimedia video consent process (and an additional US $25 at the first on-site visit at which study procedures were conducted). Thus, the payment amount was equivalent between trials for the consent substudy. Payment was issued by a check from the study site in the LASST trial and by a reloadable debit card in the MICT trial.

### Objective

The objective of the consent substudy was to compare the comprehension and retention of informed consent elements using an interactive multimedia web-based delivery of consent information (multimedia method) developed for MICT versus paper consent and assent documents and in-person face-to-face discussions with a study coordinator (conventional method) in LASST. We made comparisons between formats in the caregiver and adolescent participants. We hypothesized that consent comprehension and retention with the multimedia web-based delivery would be noninferior to the conventional in-person, face-to-face consent process.

### Interventions

#### Conventional Informed Consent Document for LASST

Parental permission and adolescent assent documents were developed according to the requirements of the IRB of the study site. Parental permission and assent were 13-page and 2-page single-spaced typed documents, respectively. The Flesch Kincaid Grade Level for the parental permission and assent were 9.8 and 6, respectively.

#### Conventional Informed Consent Process for LASST

Participants were contacted via the usual processes at the study site (telephone, provider referral, clinic intercepts, and response to flyers). Parental permission and adolescent assent were obtained through a conventional in-person interaction between the caregiver and the study coordinator and between the adolescent and the study coordinator, respectively. During the substudy training, the coordinators were instructed to maintain their usual consenting process for the LASST trial participants to avoid influencing study outcomes. The caregivers and adolescents were provided the consent documents upon arrival at the enrollment visit (or emailed or mailed to the family at their request ahead of the visit) and allowed as much time as needed, generally approximately 20 to 30 minutes, to review the document.

The study coordinator then began audio recording (Audacity) the consent process interaction for later analysis by trained coders [25]. The coordinator reviewed each section of the document with the caregiver and adolescent and answered questions. Once complete, the coordinator conducted the consent comprehension assessment and then administered the Newest Vital Sign (NVS), a health literacy tool, separately with the caregiver privately. The adolescent was then brought into the room for comprehension assessment and NVS administration [20,26-31]. As the caregiver was still in the room with the adolescent, the coordinator instructed the caregiver not to cue the adolescent with answers to the assessment questions. The coordinator reviewed responses that suggested an incomplete understanding of the study information and answered questions before obtaining caregiver and adolescent signatures on the parental permission form and assent documents, respectively.

#### Interactive Multimedia Informed Consent Video for MICT

The interactive multimedia video and website provided study information, including all the required and supplemental elements of informed consent [8]. The video storyboard and audio script were developed from the content in the 13-page parental permission form used for LASST [23]. The video had 5 sections, each 3 to 4 minutes in length, and was designed using theory and principles to facilitate electronic learning [21,22,32]. Professional video directors and professional actors were used to create the video. A segment of the video was previously published [20,21].

The video was housed within a framework in which a content-related sidebar provided additional study information by the participant selecting a tab that changed color when the video reached a relevant section on the sidebar. Each section had 2 to 3 multiple-choice questions that had to be answered before the next video section became available. The correct response was always provided to reinforce learning. The sections were programmed to be viewed sequentially to ensure that the entire video was watched.

#### Consent Process for Interactive Multimedia Informed Consent Video for MICT

Participants were contacted via telephone to participate in the MICT trial, and if interested, a private link to the interactive multimedia web-based informed consent video was sent via email to the caregiver and adolescent 4 days before a scheduled consent comprehension assessment; the 4-day period was to allow sufficient time for both the caregiver and adolescent to view the video 1 or more times. The consent comprehension assessment was conducted during a one-on-one audio-recorded call (WebEx, Cisco Systems) with the study coordinator. The audio recordings were saved for later analysis by the trained coders. The caregiver and adolescent comprehension were evaluated separately. Following the assessment, the coordinator reviewed the understanding of the study and answered any remaining questions. The NVS was then administered separately to the caregivers and adolescents. Parental permission and assent signature forms were sent via a secure patient portal from Nemours Children’s Health for electronic signatures and stored in the adolescents’ electronic health records.

### Measurements

Participants’ comprehension of the elements of informed consent was measured at enrollment upon study entry and again at study end after 20 weeks of study participation. The NVS was administered to caregivers and adolescents at enrollment only.

#### Newest Vital Sign

The NVS is validated by a 6-item survey, which measures general health literacy in children and adults and requires 3 to 4 minutes for administration [27]. The survey uses an ice cream nutrition label and incorporates reading, comprehension, and numeracy skills. The instrument was selected over other literacy assessment tools because it is a valid and reliable screening tool for caregivers and adolescents, has no ceiling effect, and can be administered in person and remotely [20,27,33,34]. The NVS was included as a covariate in the comprehension analysis.

#### Consent Comprehension Assessment Tool for LASST and MICT

The comprehension assessment tool was developed by the study psychologists and principal investigator as a 17-item open-ended questionnaire designed to assess the knowledge and comprehension of the consent material (Textbox 1). The tool was derived from questionnaires previously developed by coinvestigators in preliminary studies (NCI R03CA133442 and NCI R03CA133419; T Wysocki, PhD, unpublished data, 2010). The tool used in this study was slightly modified from the original tool by replacing 1 question that was considered redundant and adding a question on payment for participation. The psychology staff trained the study coordinators on each interview question and to use nonleading prompts to elicit further knowledge when appropriate. Each consent assessment question was scored by 2 coders as incorrect, partially correct, or correct (scored as 1, 2, and 3, respectively). The possible scores ranged from 17 to 51, with higher scores indicating better comprehension. Scores between coders were reviewed frequently early in the trial, and discrepancies between the coders were resolved by mutual agreement, with input from the principal investigator as needed, to ensure overall consistency in scoring.

**Seventeen-item questionnaire for consent comprehension assessment used for both consent formats.**

1. Please tell me the researchers’ reasons for doing this study.
2. Please tell me how much of your child’s (your) time is required while you are in this study.
3. Please tell me the main things that your child (you) will need to do at each study visit.
4. Please tell me about the study treatments that are being tested.
5. Please tell me what the chances are that your child (you) will get one kind of treatment or another.
6. Please tell me how many other people will be in this study.
7. Please tell me what bad things or risks there could be from being in this study.
8. Please tell me what the good things or benefits there are from being in this study.
9. Please tell me what other choices your child has (you have), aside from being in this study.
10. Please tell me how the researchers will protect your child’s (your) privacy while being in this study.
11. Please tell me who’s responsible for your child’s (your) medical costs if your child gets (you get) hurt or sick while your child is (you are) in the study.
12. Please tell me who you (your parent) should call if your child has (you have) questions about the study.
13. Please tell me what your child (you) should do if your child wants (you want) to stop being in the study.
14. Please tell me if and why the researchers could take your child (you) out of the study without your permission.
15. Please tell me why researchers would give your child (you) new information about this study while your child is (you are) in it.
16. Please tell me what type of payment or rewards your child (you) will get for being in the study.
17. Please tell me why your child is (you are) being asked to be in the study.

### Statistical Plan

#### Sample Size and Power

The hypothesis was that study comprehension following the novel multimedia web-based video consent process would be no worse than that following the conventional process; therefore, a noninferiority design was used. There was no expectation that comprehension would be better with the multimedia web-based consent, even with the incorporated learning principals. For ethical reasons, a noninferiority design was selected to test that the multimedia web-based consent was no worse than, or not inferior to, the conventional in-person consent process and thus is consistent with regulations governing human subjects research [1]. In addition, using a noninferiority analysis allowed for superiority to be tested if noninferiority was established. The sample size determination, using the caregiver comprehension assessment scores from preliminary data obtained in 2 R03 grants (NCI R03CA133442 and NCI R03CA133419; T Wysocki, PhD, unpublished data, 2010), assumed the normalcy of the data and defined the noninferiority margin as 2.4, which corresponds to 0.5 SD units. One-half SD was considered a clinically reasonable margin to consider the interactive multimedia web-based video noninferior to the conventional consent format and is consistent with empirical results on participant-reported outcomes [35]. On the basis of these calculations, the randomization of 120 caregivers and adolescents (60 dyads each for the multimedia web-based video consent and conventional consent process) would provide 85% power to reject the hypothesis that the multimedia web-based video consent process yields statistically significant (*P*≤.05) lower (worse) scores than the conventional process at a threshold of 2.4 units on the caregiver consent comprehension assessment scale, after allowing for approximately 10% loss to follow-up. Preliminary data indicated a greater noninferiority margin of 3.2 for adolescents. We chose the caregiver margin for sample size determination because, for a typical clinical study, caregivers largely determine whether a child will participate, and they engage in explaining the study to their child, who is then asked to provide assent.

#### Data Analysis

Data included in the analysis were from participants randomized to treatment assignments in the LASST or MICT trials. Adolescent characteristics at enrollment were compared using chi-square and Kruskal-Wallis tests for categorical and continuous outcomes, respectively. Unadjusted mean and 95% CI scores were calculated and compared between the consent processes at enrollment using generalized estimating equations. The same methods were used to compare scores at enrollment and at 20 weeks between and within each group to assess the retention of study information. The noninferiority margin was 2.4, that is, the interactive multimedia web-based video consent process was noninferior to the conventional consent process if the upper bound of the 95% CI for the difference (conventional minus multimedia website) was <2.4. The a priori margin of noninferiority was determined from the data using the caregiver consent comprehension assessment tool in the preliminary studies. The same noninferiority margin was used to evaluate adolescent scores. If noninferiority was met, the superiority of the interactive multimedia web-based video consent was evaluated using a conventional cutoff, and the 95% CI for the difference did not include 0. Exploratory univariate regression was conducted to identify the characteristics predictive of the primary outcome, followed by sensitivity analysis to determine the effect on comprehension scores. There was no controlling for multiple comparisons. The data were analyzed using SAS (version 9; SAS Institute) and R (version 4.1.2; The R Project for Statistical Computing).

## Results

### Overview

The consent substudy in the MICT and LASST trials was conducted from November 2013 to February 2017 at the 6 sites conducting both studies. These trials were conducted concurrently, and randomization across trial types was initially planned; however, rapid enrollment completion in the LASST trial across the 18 ACRC network sites resulted in 37 participants being enrolled in the LASST consent substudy rather than the 60 participants planned. In total, 71 participants were enrolled in the MICT consent substudy.

### Characteristics of Adolescent Participants

**Figure 2:**
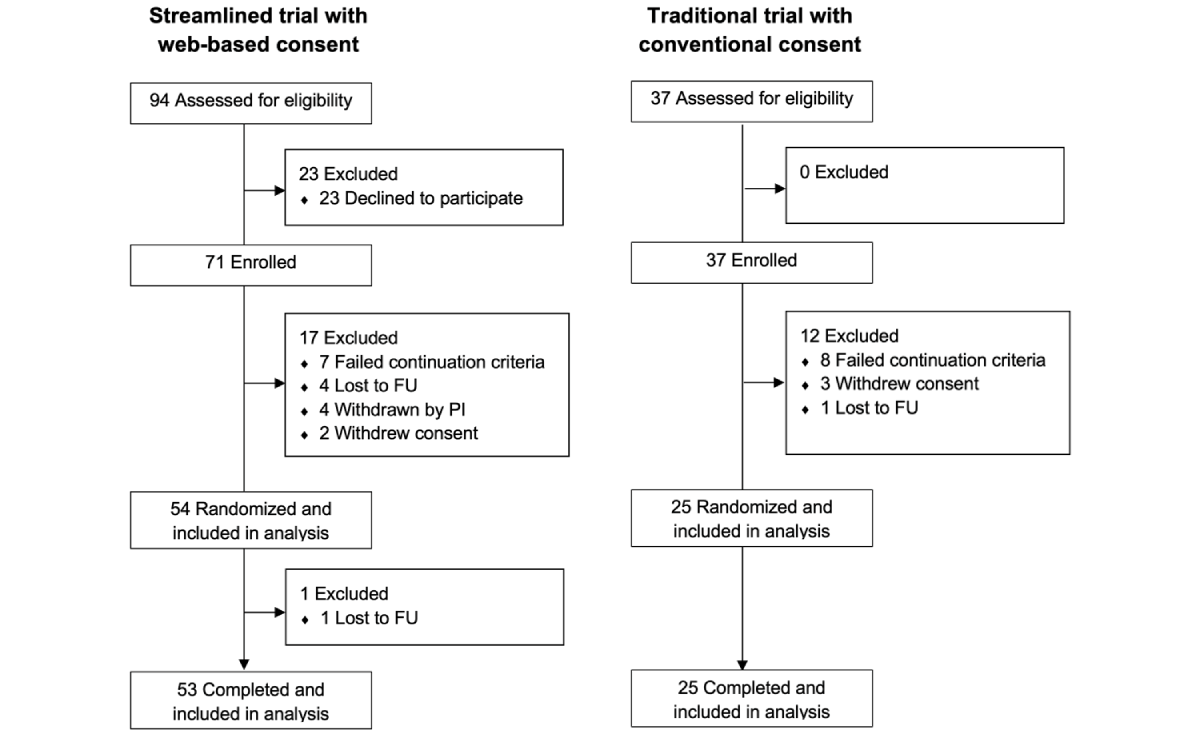
Flow of participants by trial type. FU: follow-up; PI: principal investigator.

In the trials, 108 adolescents were enrolled, of whom 79 (73.1%) were allocated to treatment and were included in the analysis (Figure 2). There were no statistically significant differences in baseline characteristics between the groups except for prebronchodilator forced expiratory volume in 1 second: the median values were 90% (IQR 80-97) predicted in the conventional group and 95% (IQR 85-107) predicted in the web-based group (*P*=.04; Table 1).

**Table 1:** Characteristics of caregivers and adolescents at enrollment by consent format.

Characteristic				Total (n=79)		Conventional consent process (n=25)		Interactive multimedia consent video and website (n=54)	
Adolescent sex, male (vs female), n (%)				45 (57)		13 (52)		32 (59)	
Caregiver sex, male (vs female), n (%)				9 (11)		4 (16)		5 (9)	
Adolescent age, median (IQR)				14 (13-15)		15 (13-16)		14 (13-15)	
Adolescent race, Black (vs non-Black), n (%)				26 (33)		10 (40)		16 (30)	
**Income (US $), n (%)**									
	≤$60,000		48 (73)		14 (70)		34 (74)		
	>$60,000		18 (27)		6 (30)		12 (26)		
	Income missing		13 (16)		5 (20)		8 (15)		
**Asthma characteristics**									
	Unscheduled health care visit for asthma in prior year (vs none), n (%)		32 (41)		9 (36)		23 (43)		
	Age of asthma onset, median (IQR)		2 (1-6)		4 (1-7)		2 (1-5)		
	Secondary smoke exposure, n (%)		15 (19)		6 (24)		9 (17)		
**Questionnaires, median (IQR)**									
	**Newest Vital Sign**								
		Adolescent score (range 0-6)^a^	5 (4-6)		5 (3-6)				5 (4-6)
		Caregiver score (range 0-6)	5 (3-6)		4 (3-6)				5 (3-6)
		Asthma Control Test (range 5-25)^b^	22 (21-24)		22 (21-23)				22 (22-24)
**Spirometry, median (IQR)**									
	Pre-BD^c^ percent predicted forced expiratory volume in 1 second		94 (85-102)		90 (80-97)		95 (85-107)		
	Pre-BD percent predicted forced vital capacity		102 (94-108)		102 (96-106)		102 (93-109)		
	Peak flow (L)		390 (323-451)		350 (290-425)		398 (343-455)		

^a^Newest Vital Sign score: 0 to 1, high likelihood of limited literacy; 2 to 3, possibility of limited literacy; and 4 to 6, almost always adequate literacy.

^b^Asthma Control Test: high scores indicate better health.

^c^Pre-BD: prebronchodilator.

### Primary Outcome: Caregiver Comprehension Assessment Score

**Figure 3:**
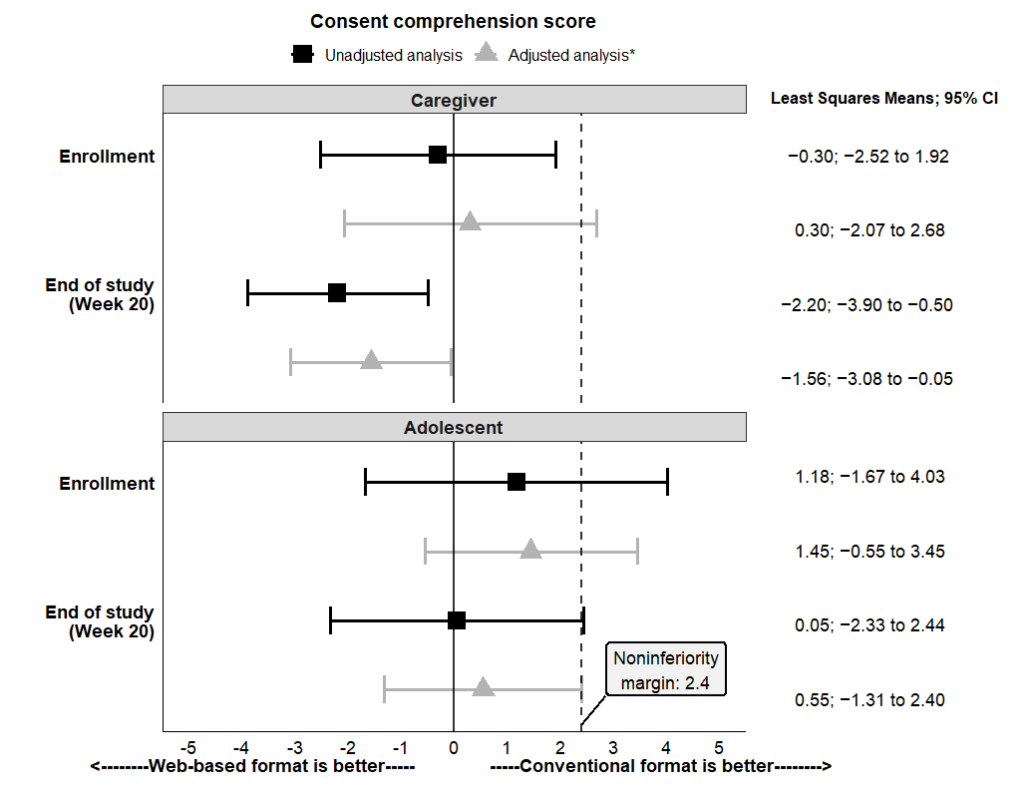
Consent comprehension score of caregivers and adolescents at enrollment and at the end of the study (20 weeks after enrollment) for the web-based and conventional consent delivery format. Noninferiority was established if the upper bound of the 95% CI for the difference was below the noninferiority margin of 2.4. Superiority of the format was determined if the noninferiority margin was met and the 95% CI for the difference did not include 0. *Analysis of scores following adjustment for baseline characteristics was not prespecified in the protocol.

The unadjusted mean (95% CI) caregiver comprehension score at enrollment was slightly higher with interactive multimedia web-based video consent (better comprehension) than with the conventional consent (Table 2) and met the criteria for noninferiority but not superiority (Figure 3). At the final study visit (20 weeks after enrollment), caregiver scores declined in the conventional group and remained stable in the multimedia web-based video consent group, such that comprehension in the multimedia web-based video consent was superior to conventional consent (Figure 3 and Table 2).

**Table 2:** Consent comprehension scores for caregivers and adolescents at enrollment and the end of the study (week 20).

			Conventional, mean (95% CI)		Web-based, mean (95% CI)
**Caregiver score**					
	Enrollment	42.90 (41.06 to 44.74)		43.20 (41.96 to 44.45)	
	End of the study (week 20)	41.08 (39.67 to 42.49)		43.28 (42.33 to 44.23)	
	Change from enrollment	−1.65 (−3.10 to −0.19)		0.14 (−0.84 to 1.12)	
**Adolescent score**					
	Enrollment	42.26 (39.90 to 44.62)		41.08 (39.48 to 42.69)	
	End of the study (week 20)	41.27 (39.29 to 43.25)		41.22 (39.88 to 42.55)	
	Change from enrollment	−0.75 (−2.27 to 0.77)		0.16 (−0.86 to 1.18)	

### Adolescent Comprehension Scores

Among adolescents, the unadjusted mean (95% CI) scores at enrollment were higher in the conventional group. The noninferiority of the interactive multimedia web-based video consent was not established because the upper bound of the CI for the difference in comprehension scores was 4.03, which exceeds the noninferiority margin of 2.4 (Figure 3 and Table 2). At 20 weeks, neither group had a reduction in comprehension, and the difference between the 2 groups of adolescents was less; however, the upper bound of the CI (2.44) exceeded the noninferiority margin.

### Exploratory Analysis

In the exploratory analysis, adolescents’ race (*P*=.009), having an unscheduled health care visit in the prior years (*P*=.02), and NVS scores for caregivers (*P*<.001) and adolescents (*P*=.03) were significantly associated with the caregiver’s score (all caregivers in both trials) at enrollment (Table 3). Adolescents’ sex (*P*=.008), age (*P*<.001), and race (*P*<.001) and both adolescents’ (*P*=.004) and caregivers’ (*P*<.001) NVS scores were significantly associated with adolescents’ scores (all adolescents in both trials) at enrollment (Table 3). These same variables were significantly associated with scores for all caregivers and adolescents at week 20, except for having an unscheduled health care visit in the prior year, which was no longer associated with scores for caregivers (data not shown). Our sample was too small to meaningfully assess whether the multimedia video consent had a greater effect on comprehension scores in those with limited health literacy (10 participants from both trials) compared with the conventional consent.

After adjustment for variables identified in the exploratory analysis (participant race, unscheduled health care visit, and caregiver NVS score), comprehension with the multimedia web-based video consent was no longer noninferior at baseline in caregivers but remained noninferior and superior to the conventional consent at 20 weeks after enrollment (Figure 3). There were no significant interactions between the predictors.

**Table 3:** Univariate association of baseline characteristic with comprehension score in caregivers and adolescents at enrollment (conventional and web-based combined).

Participant characteristics	Outcome: score at enrollment				
	Caregivers			Adolescents	
	Estimate (95% CI)	*P* value	Estimate (95% CI)		*P* value
Sex, male (vs female)	−1.10 (−3.17 to 0.97)	.30	−3.51 (−6.09 to −0.94)		.008
Age (years), anchored at 14 years	0.51 (−0.09 to 1.11)	.09	1.29 (0.56 to 2.03)		<.001
Race, Black (vs non-Black)	−2.83 (−4.93 to −0.72)	.009	−5.87 (−8.39 to −3.35)		<.001
Unscheduled health care visits for asthma in the previous 12 months before enrollment (vs none)	−2.41 (−4.45 to −0.38)	.02	−1.50 (−4.19 to 1.19)		.27
Secondary smoke exposure	−2.52 (−5.09 to 0.05)	.06	−1.26 (−4.65 to 2.12)		.47
Age of asthma onset (years)	−0.01 (−0.27 to 0.26)	.97	0.28 (−0.05 to 0.62)		.09
Per point of caregiver NVS^a^ from 0	1.06 (0.49 to 1.64)	<.001	1.14 (0.37 to 1.90)		.004
Per point of participant NVS from 0	0.70 (0.09 to 1.30)	.03	1.74 (1.03 to 2.45)		<.001
Per point in Asthma Control Test from 0	0.01 (−0.69 to 0.72)	.97	0.20 (−0.71 to 1.10)		.67
Pre-BD^b^ percent predicted forced expiratory volume in 1 second (per 10% unit difference)	0.01 (−0.74 to 0.76)	.99	0.13 (−0.84 to 1.10)		.80
Pre-BD percent predicted forced vital capacity (per 10% unit difference)	−0.71 (−1.57 to 0.14)	.10	−0.16 (−1.29 to 0.96)		.77
Peak flow (per 100 L)	0.49 (−0.55 to 1.53)	.36	0.91 (−0.42 to 2.24)		.18

^a^NVS: Newest Vital Sign.

^b^Pre-BD: prebronchodilator.

## Discussion

### Principal Findings

In caregivers, we found that comprehension with the web-based delivery format was noninferior to the conventional format at enrollment and, importantly, that study information was retained better with the web-based delivery when assessed 20 weeks later. With both consent formats in caregivers and adolescents, assessment scores indicated acceptable comprehension at enrollment (approximately 83%) out of the total possible score of 51, and there may have been little room for improvement to demonstrate the superiority of web-based delivery. We intentionally designed the interactive multimedia web-based consent using established principles for web-based learning, which may have facilitated retention. The retention of consent information over the study period with web-based delivery is noteworthy because it is essential that information about the study is understood throughout participation.

In adolescents, the score for the interactive multimedia web-based process did not meet the noninferiority margin at baseline or 20 weeks later. The caregiver score from the preliminary data was used to estimate the sample size and for the primary outcome because caregivers consider their parental permission before the child is presented with the option to assent or dissent from participation. Thus, it is essential for caregivers to have a thorough understanding of the research study to help their children have a meaningful comprehension of the presented information during the assent process. A wider margin of 3.2 from the preliminary data in adolescents may have been more appropriate because adolescent cognitive capacity and attention span are less than what is expected in adults [36]. If a margin of 3.2 was used for adolescents, noninferiority at enrollment would not have been established, although it would have been at the 20-week assessment. This finding suggests that perhaps the experiential component of trial participation aided in retention of study information at the end of the study for adolescents.

Our exploratory analysis suggests that there may be variables associated with adolescents that influence caregiver as well as adolescent consent comprehension. Unsurprisingly, health literacy scores were positively associated with comprehension scores in both caregivers and adolescents. However, larger clinical trials are required to verify the relevance of these findings.

### Comparison With Prior Work

Only 2 studies on caregivers and children have incorporated some of the features used in this project, and neither study was enrolling children in an actual clinical trial [13,14,37]. O’Lonergan and Forster-Harwood [38] evaluated study comprehension of hypothetical medical procedures (dual-energy x-ray absorptiometry and ultrasound) delivered by a multimedia PowerPoint (Microsoft Corporation) presentation with video hyperlinks designed with a “learning objective approach” versus a standard IRB templated paper document in caregivers and young adolescents. Both caregivers and adolescents had better comprehension of the medical procedures with the PowerPoint presentation assessed by semistructured interviews. Unlike our study, the caregivers and adolescents read the paper document of the medical procedures without contemporaneous review of content with a study staff member before assessment, which may have biased comprehension in favor of the PowerPoint presentation.

Tait et al [39] developed a presentation of pictorials and touch-and-drag features to explain research concepts (eg, randomization and blinding) with voice-over on an iPad and compared comprehension with paper-based explanations; the material was not for a clinical trial. Parents demonstrated no difference in comprehension between formats when assessed by semistructured interviews, whereas children had greater comprehension after viewing the iPad pictorials. As with the study by O’Lonergan and Forster-Harwood [38], there was no review of the paper-based material with the parent or child before the interview, which may have influenced the better comprehension scores for the iPad presentation.

In a review of strategies designed to improve consent comprehension through various methods, including multimedia processes, Abdel-Rahman [13] found that recall and comprehension are enhanced by formats that include both audio and video components, even without an interactive component [40]. An evaluation of digital tools for informed consent found that multimedia formats (images, audio, videos, and graphics) had a greater impact on understanding, satisfaction, anxiety, and participation compared with videos (audiovisual) only, perhaps because videos do not enhance information already communicated through in-person interaction [37]. Improvements with multimedia formats have tended to be modest, which may be a function of the characteristics of the format as well as the method for assessing comprehension [13].

In this study, we assessed comprehension using open-ended questions that were graded by a team of trained scorers. A systematic review found that the understanding of study material assessed by closed-ended questions was better than that assessed by open-ended questions [14]. Open-ended questions, as in semistructured interviews, require the comprehension of previously stored information that is more difficult than responding to closed-ended questions, such as multiple choice, which rely on the recall of a correct response out of presented possibilities. The semistructured interviews used in this study elicited a more complete assessment of the learned material.

This is the first study to test consent information comprehension in caregivers and adolescents who experienced an interactive multimedia web-based video consent format versus a conventional written consent document with in-person face-to-face interaction in an actual asthma clinical trial that enrolled adolescents. Our intent was to determine whether interactive multimedia web-based video consent provided clinical trial information and promoted understanding of that information in a manner that was similar or noninferior to a conventional in-person, face-to-face consenting process that included a review of the consent of a study staff member. Our study has important findings that can guide the development of interactive multimedia web-based consent formats for future clinical trials. The platform was intentionally developed using 5 specific theoretically grounded principles of multimedia learning, with an intentional focus on appeal to people with low health literacy [21]. In addition, the quizzes embedded in the consent video reinforced the key study information. We used a semistructured interactive interview to assess and score comprehension at enrollment and again 20 weeks later to assess the retention of study information rather than multiple-choice or true-false questions. This is one of the few studies to address modifying the assent process to improve comprehension in minors [19].

### Limitations

Our study has several limitations. First, enrollment by trial type was not randomized as initially intended because of the rapid pace of participant accrual into the conventional trial conducted across the entire network of study sites. As a result, the enrollment goals were not met. However, with the available sample, we were able to demonstrate noninferiority for the interactive multimedia web-based video consent format at enrollment and superiority at study end compared with the conventional consent process in caregivers. Second, we did not have an established noninferiority margin specified for adolescents, and we did not establish noninferiority using the specified margin. However, in pediatric trials, the caregiver is largely the decision maker, with assent or dissent provided by the child participants; thus, the comprehension assessment by the caregiver was considered of principal importance for sample size estimation and outcome analysis. Third, it is possible that the study coordinator’s knowledge of the comprehension score being measured may have altered their typical consenting style (known as the Hawthorne effect) to be more thorough or engaging, thus resulting in higher scores for the caregiver and adolescent with the conventional consent format [41]. The coordinators were instructed at the study outset to maintain the consent process used in other clinical trials. In contrast, there was no coordinator interaction with participants who received the web-based format to facilitate comprehension before comprehension assessment. It is possible that the focus on the consent process may have preferentially favored the conventional consent format, thus reducing the score differences. Fourth, this was a pilot study comparing consenting formats in a population with a chronic disease and may not be applicable to patients with cancer or acute illnesses; however, our results need to be replicated in other intervention trials that include children and adolescents with chronic conditions (eg, Crohn disease, allergies, and epilepsy).

It is important to acknowledge that the development of the video and website required resources (videographers, actor talent, website designers, and psychologists) that may not be readily available at an institution planning a small clinical trial. Clinical trials are expensive to perform, with costs for study procedures as well as for less apparent costs of time and effort associated with patient recruitment and retention [42]. Pediatric clinical trials are particularly labor intensive and expensive to perform, and joining a trial is often perceived as inconvenient and time-consuming by families [43-47]. Parents often have to bring siblings to appointments and manage competing after-school priorities to travel to a study site. The interactive multimedia video consent and website were developed for the MICT trial to evaluate the consenting process via the internet to reduce the burden of having an on-site study site visit for the purpose of learning about the study before considering enrollment. Failure to recruit participants can extend trial completion and thus costs or result in premature trial discontinuation and wasted resources [42]. Therefore, how to effectively spend the limited resources that are needed for recruitment materials, stipends, and retention incentives requires careful consideration that may be study specific or site specific. The development of a video consent with the resources used in this trial may be best suited for large multisite trials; those conducted by the pharmaceutical industry; grants in which funding can be included in a budget proposal; or trials governed by a single IRB process, which is becoming more common. We found that the trial information was effectively conveyed and understood with the multimedia web-based consent designed for this trial.

Looking forward, advancements in artificial intelligence to create or manipulate multimedia content for film production in the absence of videographers and live actors may overcome some of these barriers [48]. Currently, there are software programs geared toward nonprofessionals to create simplified animated videos (Doodly, CreateStudio, and Cinema 4D) that may be suitable for creating consent documents or supplemental consent information [49]. The development of this multimedia platform would not have been possible without considerable dialogue with the IRB of the lead institution.

### Conclusions

The new findings resulting from these data are that the comprehension of trial information can be effectively communicated with an asynchronous interactive multimedia web-based consent developed with established principles of learning for caregivers of adolescents with asthma. Embedding these learning principles may aid in the retention of study information over the duration of the study, as we found in this trial. We do not suggest that a participant’s trial participation decision should occur in the absence of a discussion with the study staff. However, avoiding a long on-site study appointment to simply learn about the study before deciding to participate may relieve some of the burden of study participation for caregivers and their children and the study staff. Thus, modernizing the consent process to convey necessary clinical trial information in a manner that promotes improved comprehension across ages and sociodemographic groups is essential. Finally, the COVID-19 pandemic has had a substantial impact on the conduct of on-site study visits with participants and institutions alike, redirecting in-person, face-to-face study visits to telehealth. Thus, greater attention to strategies to conduct research in study site–independent venues will likely be a critical consideration in the design of future clinical trials to facilitate trial enrollment.

## Abbreviations

ACRC: Airways Clinical Research Centers
IRB: institutional review board
LASST: Long-Acting Beta-Agonist Step-Down Study
MICT: Use of Mobile Devices and the Internet to Streamline an Asthma Clinical Trial
NVS: Newest Vital Sign

## Data Availability

The data sets generated and analyzed during this study are available from the corresponding author on reasonable request.
